# Clinical effectiveness of family therapeutic interventions in the prevention and treatment of perinatal depression: A systematic review and meta-analysis

**DOI:** 10.1371/journal.pone.0198730

**Published:** 2018-06-14

**Authors:** Fallon Cluxton-Keller, Martha L. Bruce

**Affiliations:** 1 Department of Psychiatry, Geisel School of Medicine at Dartmouth, Lebanon, New Hampshire, United States of America; 2 General Pediatrics, Johns Hopkins School of Medicine, Baltimore, Maryland, United States of America; Stanford University School of Medicine, UNITED STATES

## Abstract

**Background:**

Family therapy is a potential strategy to increase family support for those suffering from perinatal depression. Family therapeutic interventions for this population typically target depressed women and their adult family members to improve family functioning and reduce depressive symptoms.

**Objective:**

This systematic review and meta-analysis is a synthesis of the current evidence on the usefulness of family therapy interventions in the prevention and treatment of perinatal depression and impacts on maternal depressive symptoms and family functioning.

**Methods:**

This study used the Cochrane Collaboration guidelines for systematic reviews and meta-analyses. Six electronic databases were searched for randomized controlled trials and cluster randomized trials. The primary outcomes included maternal depressive symptoms and family functioning.

**Results:**

Seven studies were included in the qualitative and quantitative analyses. Fixed effects models showed statistically significant reductions in depressive symptoms at post-intervention in intervention group mothers. Intervention intensity and level of family involvement moderated intervention impacts on maternal depression. A fixed effects model showed a trend in improving family functioning at post-intervention in intervention group couples.

**Conclusion:**

Although a limited number of controlled trials on family therapeutic interventions for this population exist, the findings show that these types of interventions are effective in both the prevention and treatment of perinatal depression. Recommendations for future research are addressed.

**Systematic review and meta-analysis protocol registration:**

PROSPERO, CRD42017075150.

## Introduction

Perinatal depression has become a growing problem worldwide. Depressive symptoms develop anytime during pregnancy or within the first year after childbirth [1]. In the United States, about 6% of women experience depression during pregnancy [2] and one in nine women experience postpartum depression [3]. The World Health Organization (2017) reported that in developing countries, nearly 16% of women experience depressive symptoms during pregnancy and nearly 20% experience depressive symptoms after childbirth [4].

Research has shown that untreated perinatal depression can adversely affect birth outcomes [5–6], result in poor maternal-infant interaction [7–8] and increase the risk for child maltreatment [9]. For these reasons, many professional organizations (e.g., American College of Obstetrics and Gynecology, American Academy of Pediatrics) have recommended the use of depression screening measures in primary care settings and raised awareness of the need for treatment. The majority of preventive and treatment studies for perinatal depression include individual-level interventions targeting only mothers [10–11] or interventions that target maternal-infant interaction [12–13].

Research has identified a bidirectional association between family relational stress and perinatal depression in that lack of family support is both a predictor [14–15] and a consequence of perinatal depression [16]. Frequent arguments [17], gender role stress (e.g., males expression of fears of performance failure related to work and sex, female expression of fears of disruption in the couple relationship due to the introduction of a child) [18], conflict because one or both partners did not want the pregnancy [19], division of labor [20], poor support following stressful life events [21], lack of partner availability [22], and low intimacy [23–24] are associated with increased perinatal depressive symptoms. Several studies have also shown that marital/relationship dissatisfaction is associated with perinatal depression [16–17, 25–28]. For example, one study compared depressed and non-depressed women at two months post-delivery and found that women with depressive symptoms perceived that their partners did not share similar interests, provided little companionship, expressed disinterest in infant care, did not provide a feeling of connection, did not encourage them to get assistance to cope with difficulties, and expressed disagreement in infant care [16].

Family therapy interventions aim to change family dynamics, such as those mentioned in the above paragraph. Family therapy is based in systems theory, which generally focuses on the ways in which family interactions affect each individual family member’s functioning and affect the family’s overall functioning [29–30]. In considering the effectiveness of family therapy for treatment of depression outside the perinatal period, studies have shown that family therapy is an effective treatment for youth and adults with only depression and those with co-occurring depression [31–33].

Over the past decade, a growing body of research on the effectiveness of family therapeutic interventions for the prevention and treatment of perinatal depression has emerged. Research has shown that family therapeutic interventions that aim to prevent or reduce perinatal depression target communication skills related to expectations (including those that pertain to gender roles and the transition to parenthood) and emotional support [34–40], conflict management [38–39], and problem-solving skills related to shared responsibility in infant care and household activities [36–39]. Researched interventions that target these areas are usually theoretically based in psychoeducational [34] or cognitive-behavioral family therapy models [36–40].

Although perinatal depression affects family relational health [16], few high quality studies on psychotherapeutic treatments include family therapeutic interventions [34–35].The scope of preventive interventions that target mothers with subclinical depressive symptoms has greatly expanded over the past decade. Although most preventive psychotherapeutic interventions target only mothers with perinatal depressive symptoms [11], a growing awareness has developed regarding the importance of including the mothers’ adult family members in interventions that aim to reduce perinatal depressive symptoms [40]. For example, there are systemic interventions for couples that focus on the transition to parenthood and aim to prevent the development of perinatal depression [36–40]. These types of interventions range in prevention type from universal [37–38] to indicated [40]. Although these types of interventions target couples, the focus is generally to improve communication to strengthen parenting [37–38,40]. Some of the general goals of transition to parenthood programs are to facilitate healthy communication between parents, develop healthy family rules and limits, create shared responsibility in childrearing, and to teach parenting skills to increase positive behavior and prevent misbehavior in children [36–38].

### Current study

The purpose of the current study is to evaluate the existing evidence on the effectiveness of family therapeutic interventions in reducing perinatal depressive symptoms and improving family functioning. We broadly defined family functioning as relationship satisfaction, cohesion, and couple communication as it pertains to emotional expression and problem-solving. In our study, we defined family therapeutic interventions as those that aim to improve relationships between the mother and at least one adult family member. These interventions included in this review are systemic in nature and address relationship dynamics (e.g., cohesion, couple communication) to create change to improve relational health.

The protocol for the current systematic review and meta-analysis is registered with PROSPERO (CRD42017075150). We acknowledge that the methods of our study can be compared and contrasted to those of other similar review studies [10–11, 13, 41–48]. The current study shares four similar methods with the previously published systematic reviews and meta-analyses. First, the interventions examined by us and others [10–11, 13, 41–43, 46] primarily focus on preventive psychosocial interventions or psychosocial treatments that aim to reduce perinatal depressive symptoms. Second, primary outcomes include both maternal depressive symptoms and family functioning [10, 13, 11, 41–43, 45–46]. Third, we conduct separate analyses for treatment and prevention studies using data analytic strategies that are similar to those in other published reviews [11,43]. Finally, our study and other studies [11, 42] only include randomized controlled trials and cluster randomized trials.

The methods of our study differ from the other published reviews on similar topics [10–11, 13, 41–42, 45–46] in two ways. First, we have only included studies with family-based interventions that aimed to prevent or treat perinatal depression *and* improve family functioning. Second, we assess level of family involvement as a moderator of intervention impacts on maternal depressive symptoms.

In summary, our study will enhance the existing knowledge on effective family-based psychotherapeutic interventions for the prevention and treatment of perinatal depression. This study has three aims in the provision of preliminary evidence to: 1) extend existing clinical recommendations; 2) define a general level of family involvement that is necessary for mothers to achieve a reduction in depressive symptoms; and 3) define the appropriate intervention dose in which families achieve intended outcomes.

### Objectives

This study seeks to answer the following research question: What evidence exists on the effectiveness of family therapeutic interventions for the prevention and treatment of perinatal depression? We hypothesized that intervention impacts on maternal depressive symptoms and family functioning would be moderated by intervention intensity (prevention versus treatment), level of family involvement (average number of intervention sessions attended by mothers and their adult family members), and dosage (average session length and number of sessions attended by mothers). Our study explores the impact of these moderators on outcomes. Although the included studies that used different intervention models and included family therapeutic interventions from different theoretically-based models, variation in theoretically-based family therapeutic interventions used in the experimental groups only existed between the two treatment studies included in our review. All of the prevention studies (n = 5) included in our review used family therapeutic interventions that were theoretically based in cognitive-behavioral models in the experimental groups. Since moderation analysis could only be performed with a minimum of five studies, we were unable to test the models in which the family therapeutic interventions were based as a moderator for the two treatment studies. The primary goal of our study is to increase awareness of the benefits of family therapeutic interventions as both preventive and treatment mechanisms for this vulnerable population and their families.

## Materials and methods

The protocol for the current systematic review and meta-analysis is registered with PROSPERO (CRD42017075150). This review included studies of family therapeutic interventions involving depressed women (pregnant *and* post-delivery) and at least one adult family member. In the current study, family member is defined as the depressed woman’s significant close other (i.e., partner/spouse, grandparent, adult sibling, etc.). A two-stage review process was used for eligible studies; first, the quality of the research design was assessed in each study and second, threats of bias were assessed [49]. The guidelines established by the Cochrane Handbook for Systematic Reviews of Interventions [50] were used in our rigorous evaluation of the included studies and our meta-analysis. Compliance with the Preferred Reporting Items for Systematic reviews and Meta-Analysis [51] guidelines was ensured.

### Eligibility criteria

We used the Population, Intervention, Comparator, and Outcome (PICO) method [52] to develop the eligibility criteria for the included studies.

#### Types of studies

The inclusion criteria encompassed randomized controlled trials and cluster randomized controlled trials testing the impact of family therapeutic interventions on maternal depression and family functioning outcomes. All studies were published in peer-reviewed journals.

#### Types of populations

Studies targeting primaparous and multiparous women were included. Studies that included the mother (pregnant and postpartum up to six months) and at least one adult family member were included. We defined family member as someone with whom the mother was biologically related (e.g., her own mother) or was her close significant other (e.g., partner or spouse).

#### Types of interventions

The studies that met inclusion criteria included systemic interventions applied to both parents/primary caregivers who were either expecting a baby or who had at least one infant. These interventions could address co-parenting, couple relational dynamics, or dynamics involving extended family members/ next of kin. All types of intervention intensity (e.g., universal, selective and indicated prevention, treatment) were included in our searches. We defined family therapy interventions as those that addressed relationship dynamics to create changes in relational functioning. Examples of family therapeutic interventions include: behavioral marital therapy, cognitive-behavioral skills training (e.g., couple communication, problem-solving, conflict management), interpersonal therapy, and solution-focused therapy.

#### Types of providers

We did not restrict our search criteria to interventions that were only delivered by mental health professionals. The majority of the studies that met the inclusion criteria included interventions that were delivered by mental health professionals (e.g., social workers, doctoral trained Licensed Marriage and Family Therapist, psychologists, psychiatrists). A few studies included interventions that were delivered by psychology trainees who were supervised by licensed psychologist and interventions delivered by trained maternal and child health nurses.

#### Types of comparators

Experimental intervention groups were compared to control groups comprised of standard care, treatment as usual, wait-listed, or no care conditions.

#### Types of outcome measures

All included studies listed maternal depression as a primary outcome and listed family functioning as either a primary or secondary outcome. The primary outcome was maternal depressive symptoms, and studies that measured change in maternal depressive symptoms from baseline to at least one post-intervention time point were included. We also included studies with measures of family functioning at baseline and at least one post-intervention time point.

### Excluded studies

The following studies were excluded from this review and meta-analyses: non-randomized trials; studies without maternal depression as a primary outcome; studies without a family functioning measure; studies focused on addiction or substance misuse/abuse; studies of mothers with medical conditions; studies of infants with medical conditions or complications at birth; studies that focused on only the mother-infant dyad without any other adult family member participation; studies of parents of children age two years old and older; studies with trauma-based interventions; studies with domestic violence interventions; studies that were not written in English; studies not published in peer-reviewed journals; and studies published prior to 1995.

### Study selection criteria

A Johns Hopkins Medical Institution Librarian expert assisted with the search syntax for each of the following six electronic databases: PubMed, PsycINFO, EMBASE, CINHAL, SCOPUS and The Cochrane Library (Cochrane Database of Systematic Reviews, Cochrane Register of Controlled Trials CENTRAL, Cochrane Methodology Register). The search syntax for each database was pilot tested, and the syntax was further refined until finalized. The initial searches were conducted from July 2017 through August 2017. The searches were re-run in each database in November 2017 immediately before the final analyses for retrieval of any new studies that met the study inclusion criteria. The searches included publication date restrictions and an English language restriction. The searches returned articles published between 1995 and 2017. The search strategy used for PubMed is included, below.

((((randomized controlled trial[pt] OR controlled clinical trial[pt] OR (randomised[tiab] OR randomized[tiab]) OR placebo[tiab] OR “drug therapy”[Subheading] OR randomly[tiab] OR trial[tiab] OR groups[tiab]) NOT (“animals”[MeSH Terms] NOT “humans”[MeSH Terms])) AND ("psychosocial”[tw] OR “psychotherapy”[tw] OR “Psychotherapy”[MeSH] OR “family therapy”[tw] OR “Family Therapy”[MeSH] OR “family intervention”[tw] OR “family-based intervention”[tw] OR “family interventions”[tw] OR “family based”[tw] OR “family systems”[tw] OR “couple therapy”[tw] OR “marital therapy”[tw] OR “co-parenting”[tw] OR “Perinatal Mood Disorders”[tw] OR “mood disorder”[tw] OR “depression”[tw] OR “Depression”[MeSH] OR “Depressive Disorder”[MeSH] OR “Depressive Disorder”[tw] OR “Depressive Disorders”[tw] OR “Depression, Postpartum”[tw] OR “Postpartum Depression”[tw] OR “postnatal depression”[tw] OR “Antenatal Depression”[tw] OR “prenatal depression”[tw] OR “Peripartum Depression”[tw] OR “Perinatal Depression”[tw] OR “puerperal depression”[tw] OR “Depressive Disorder, Major”[MeSH] OR “Dysthymic Disorder”[MeSH] OR “Dysthymic Disorder”[tw] OR “dysthymia”[tw]) AND (“families”[tw] OR “Family”[tw] OR “Family”[MeSH] OR “Parents”[tw] OR “Parents”[MeSH] OR “Parent”[tw] OR “parental”[tw] OR “parenting”[tw] OR “caregiver”[tw] OR “caregivers”[MeSH] OR “caregivers”[tw] OR “care giver”[tw] OR “care givers”[tw] OR “Spouses”[MeSH] OR “spouses”[tw] OR “spousal”[tw] OR “partners”[tw] OR “partner”[tw]) AND (“Pregnancy”[MeSH] OR “pregnancy”[tw] OR “pregnancies”[tw] OR “prenatal”[tw] OR “postpartum”[tw] OR “antenatal”[tw] OR “postnatal”[tw] OR “peripartum”[tw] OR “puerperal”[tw] OR “perinatal”[tw] OR “primiparous”[tw] OR “multiparous”[tw])) AND ("1995/01/01"[PDat]: "2017/12/31"[PDat])) Filters: Publication date from 1995/01/01 to 2017/12/31; English

The selection of studies was conducted in three stages [49]. First, a content expert (first author) screened titles and abstracts of references retrieved from the searches using the Population, Intervention, Comparator, and Outcome (PICO) method [52]. Second, the content expert independently reviewed full-text articles. Any uncertainties regarding the inclusion of articles were resolved by consensus between the content expert and an expert evaluator (second author). Finally, the content expert repeated the searches to screen titles and abstracts of references retrieved to determine if any new studies had been published that were eligible for inclusion.

### Data extraction

The content expert extracted data that aligned with the PICO criteria [52], baseline and follow up sample sizes, means, standard deviations, confidence intervals, and standard errors by study group for the primary outcome measures for all of the included studies. Demographic data were extracted for families (e.g., maternal age, paternal age, number of pregnancies, number of children, marital status, level of education, annual income) by study group. Data on the characteristics of the intervention group condition (e.g., intensity, types of systemic interventions experimental group model, number and length of sessions, duration of intervention, level of family member involvement) and control group conditions were extracted. Process data were extracted on the measurement of intervention fidelity, if reported in the article. Data were extracted to assess risks of bias and study quality (see the “Qualitative analysis of study quality” subsection for details).

## Analysis

### Qualitative analysis of study quality

Two evaluators (first and second authors) independently assessed the risk of bias for each included study using the Cochrane Collaboration's Tool for Assessing Risk of Bias, which measures six biases across seven domains: 1) Selection: random sequence generation and allocation concealment; 2) Performance: blinding of study participants and study personnel; 3) Detection: blinding of outcome assessment; 4) Attrition: incomplete outcome data; 5) Reporting: selective outcome reporting; and 6) Other: other sources of bias [53]. This tool provides detailed instructions for how to rate each risk of bias within each of the seven domains and each domain is rated as Low, Unclear, or High risk [53]. Bias in cluster randomized trials was assessed using additional criteria: 1) recruitment bias as it pertains to the randomization of all study sites at the same time; 2) baseline imbalance as it applies to the stratification or pair-matched randomization of sites; 3) explanations as to why any study sites were excluded from the analysis; 4) incorrect analysis; and 5) comparability with traditional RCTs [54]. We only included cluster randomized trials with low risk of bias these five additional criteria in the meta-analysis.

The Cochrane Collaboration Grades of Recommendation, Assessment, Development, and Evaluation [55] system was used to rate the overall quality of the seven included studies. GRADE includes a thorough assessment of the study design and its execution, consistency of the results, directness of evidence, precision, and publication bias [55]. Two evaluators (first and second authors) rated each included study on each criterion. Disagreements were resolved by consensus. Publication bias was not assessed since it can only be tested when a minimum of 10 studies are included in the meta-analysis to ensure there is adequate power to detect significant asymmetry [56]. Review Manager 5.3 software was used to create the risk of bias graphs within and across studies [57].

### Quantitative data analysis

Seven studies (five prevention studies and two treatment studies) were included in the qualitative analysis. All primary outcomes were continuous. Stata 15.1 [58] statistical software for meta-analyses was used to analyze data for the seven remaining included studies. About 57% (4/7) of the included studies measured the outcomes immediately following the final experimental intervention session, and the remaining 43% (3/7) of the studies measured the outcomes within five months of the final experimental intervention session (see *Table 1* for more details). The means and standard deviations were standardized using Stata 15.1 [58]. Standard deviations were pooled using a very conservative formula that assumed the baseline and post-intervention measures were not correlated since these were not reported in the included studies [49]. Standardized mean differences (SMD) were calculated as the bias-adjusted difference using Hedge’s g between the study groups [49]. The I^2^ statistic was used to assess heterogeneity between studies. Fixed effects models were used for the primary outcomes.

**Table 1:** Characteristics of included studies.

Study	Intervention Group Condition			Control Group Condition	Primary Outcome Measures
	Intensity, Model, and Systemic Interventions (SI)	Target Population	Intervention Duration, Session Length, and Format		
Daley-McCoy et al., 2014^e^ [20]	Intensity: Universal. Model: “Psychoeducation.” SI: Communication and problem-solving skills training^a^	Women and their partners (n = 47) expecting their first baby	Duration: 5 standard antenatal care classes with an additional session after the last class. Length: 2 hours. Format: Group.	Standard care (n = 36)	Depression: Edinburgh Postnatal Depression Scale (EPDS). Family Functioning: Couple Communication Scale.
Feinberg & Kan, 2008^b^ [22]	Level: Universal. Model: “Family Foundations.” SI: Communication, problem-solving, and conflict management skills^a^	Women and their partners (n = 79) expecting their first baby	Duration: 4 prenatal classes and 4 postnatal classes. Length: 2 hours. Format: Group.	No treatment control condition (n = 73)	Depression: Center for Epidemiological Studies Depression Scale. Family Functioning: Video-taped couple interactions.
Fisher et al., 2016^b^^,^ ^f^ [21]	Level: Universal. Model: “What Were We Thinking.” SI: Communication, conflict management skills & parenting skills^a^	First-time parents (n = 187) of infants	Duration: 1 session integrated into standard primary care program. Length: 6 hours. Format: Group.	Standard care (n = 177)	Depression: Patient Health Questionnaire– 9 item. Family Functioning: Intimate Bonds Measure and a single item relationship quality measure.
Gambrel & Piercy 2015 [23]	Level: Universal. Model: “Mindfulness-based relationship education.” SI: Cognitive, interpersonal mindfulness skills^a^	Women and their partners (n = 32) expecting their first baby	Duration: 4 weekly sessions. Length: 2 hours. Format: Group.	Wait-list control group (n = 34)	Depression: Depression Anxiety Stress Scale–Depression subscale. Family Functioning: Couple Satisfaction Index.
Misri et al., 2000 [16]	Level: Treatment. Model: “Psychoeducation.” SI: Supportive communication.	Postpartum women^g^ diagnosed with MDD^c^ and their partners (n = 32)	Duration: 6 weekly sessions followed by 1 session, a month later. Partners attended 4 of the 7 sessions. Length: Not specified. Format: Individual couple sessions.	Psychoeducation sessions with only patients (n = 25)	Depression: EPDS. Family Functioning: Dyadic Adjustment Scale.
Mulcahy et al., 2010^b^ [17]	Level: Treatment. Model: “Interpersonal Group Therapy.” SI: Partners learned to support and respond to depressed women.	Postpartum women^g^ (n = 23) diagnosed with MDD^c^ and their partners	Duration: 8 weekly sessions and 1 partner session. Length: 2 hours. Format: Group.	Treatment as usual (n = 27)	Depression: EPDS. Family Functioning: Dyadic Adjustment Scale.
Ortiz Collado et al., 2014^e^ [26]	Level: Indicated prevention. Model: Humanistic and cognitive-behavioral.^d^ SI: Communication skills to strengthen affective bonds.^a^	Pregnant women^g^ and their partners (n = 138)	Duration: 10 weekly sessions. Length: 2 hours and 15 minutes. Format: Group.	Standard Care (n = 116)	Depression: EPDS. Family Functioning: Dyadic Adjustment Scale.

^a^We classified these interventions as cognitive-behavioral.

^b^ Only included in the meta-analysis for the depression outcome. See the subsection “Managing missing quantitative data” for more details.

^c^MDD = Major Depressive Disorder.

^d^Classification of systemic interventions was confirmed through personal communication with Dr. Ortiz Collado.

^e^Follow-ups occurred between one month and six weeks (n = 2) after the last intervention session.

^f^Follow-up occurred five months after the last intervention session.

^g^Primaparous and multiparous women were included.

Random and fixed effects models were used for the subgroup analyses. The likelihood of type one and type two errors increases as more subgroup analyses are performed [59] and for this reason, subgroup analyses were only conducted on a minimum of five studies to determine if hypothesized moderators strengthened intervention impacts on outcomes. Only one subgroup analysis that included all seven studies, and the remainder of the subgroup analyses were restricted to the five prevention studies. We used a random effects model to assess whether intervention intensity (universal prevention, indicated prevention, or treatment) moderated impacted on maternal depression outcomes for all seven studies. We used stratified analysis for study quality to assess discrepancies in results that were based on bias.

We only conducted the moderation analysis using the five prevention studies. This decision was made given the differences in the sample characteristics between the two types of studies (i.e., the treatment studies only included mothers diagnosed with Major Depressive Disorder and the prevention studies included mothers with varied levels of depressive symptoms) and the focus of the intervention (i.e., treatments focused on depression and preventive interventions primarily focused on general functional improvement during the transition to parenthood). The following moderators were tested in the five prevention studies: level of family involvement in intervention sessions (attendance at least 80% of sessions vs attendance at 79% or fewer sessions) and dosage (six or more sessions totaling at least 12 hours vs five or fewer sessions under 12 hours). Although we intended to assess the quality of the monitoring of fidelity, there were too few studies (n = 4) that included information on fidelity.

### Managing missing quantitative data

For the trials that did not adjust for clustering, the recommended intraclass correlation (ICC) of 0.05 was used to calculate the design effect and to adjust the standard errors [54]. We determined that one cluster randomized trial and one randomized controlled trial (n = two prevention studies) could not be included in the analysis for the family functioning outcome because the measures could either not be standardized or the outcome was measured at time points that did not align with the measurement points in the other included studies. We also excluded one treatment study that was a randomized controlled trial from this analysis because the family functioning measure was only administered to mothers and not their family members.

## Results

**Fig 1:**
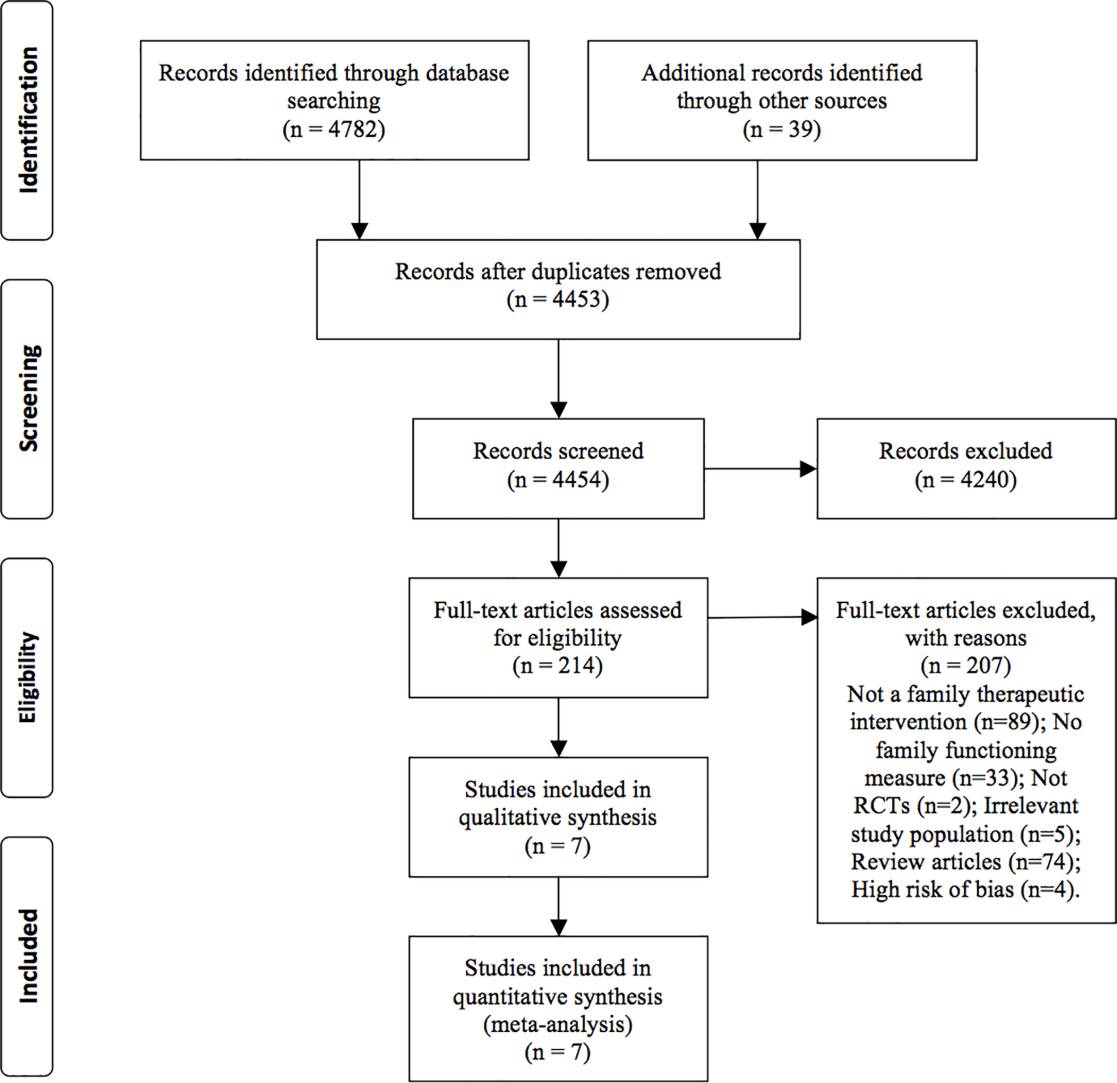
PRISMA diagram of study selection process.

Fig 1 summarizes the study selection process and adheres to PRISMA guidelines [60]. The PRISMA checklist is included in S1 Table.

### Qualitative results

A total of seven studies (n = two cluster randomized trials, n = five randomized controlled trials) were included in the qualitative synthesis. We used stratified analysis for study quality to assess discrepancies in results that were based on bias in the prevention studies and in the two treatment studies. These analyses did not produce any findings that indicated that the results were influenced by bias. The characteristics of the seven studies included in the qualitative synthesis are summarized in Table 1 by reference, intervention group condition, control group condition, and outcome measure.

As shown in Table 1, most of the studies included interventions that are based in cognitive-behavioral couple therapy. This model of therapy focuses on challenging thoughts that interfere with healthy communication and problem-solving and teaching couples specific skills to improve communication, reduce conflict, and increase shared problem-solving [61]. One study used a psychoeducational model where partners were educated on perinatal depression and positive dyadic interaction was encouraged [49]. Another study used an interpersonal model that included specific strategies for couples to increase perspective-taking, support and responsiveness [35].

**Fig 2:**
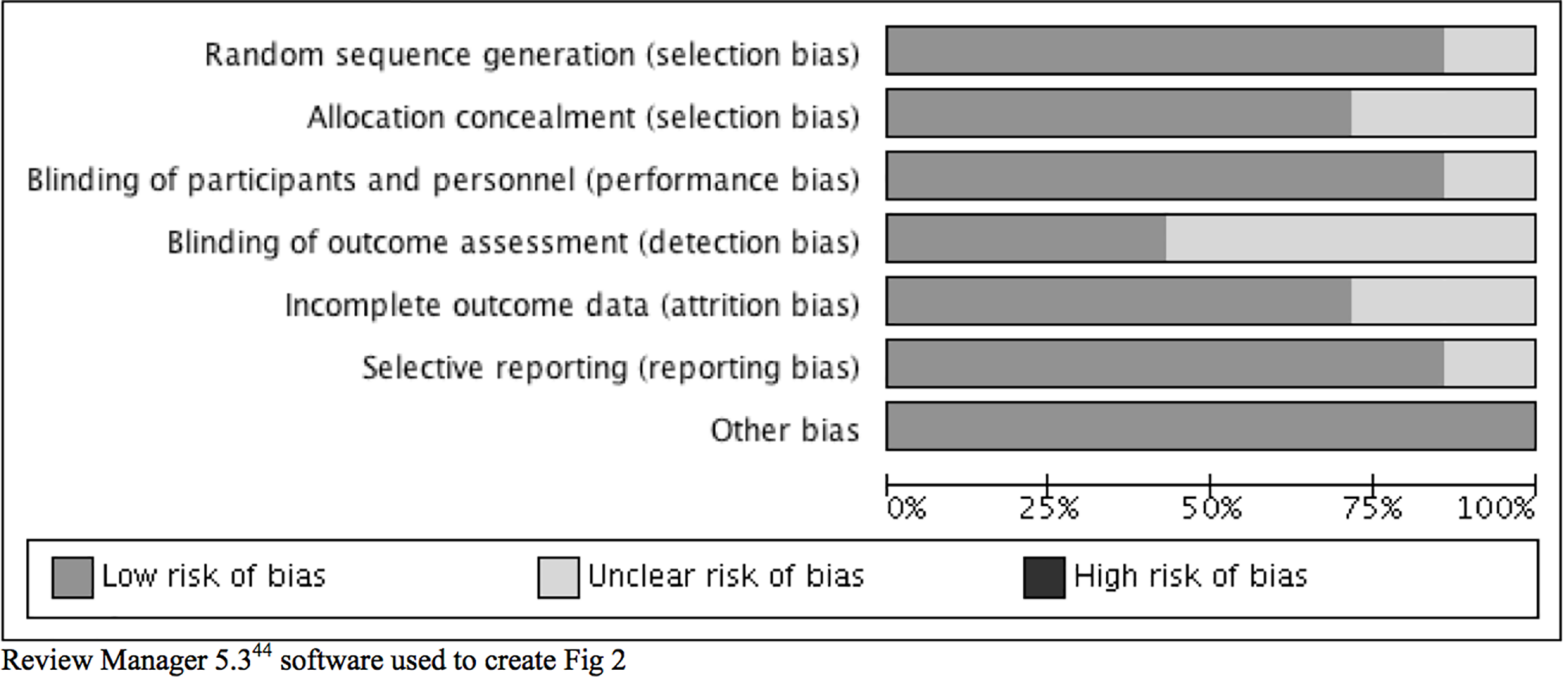
Authors’ judgments of risk of bias across all included studies. Review Manager 5.3^44^ software used to create Fig 2.

Fig 2, which summarizes the evaluators’ judgments about each risk of bias item presented across all included studies. The findings for the biases by which the seven studies were judged are summarized in the following six subsections. Review Manager 5.3 software was used to create the risk of bias graph (Fig 2) [57].

Selection bias includes both random sequence generation and allocation concealment [56]. As shown in Fig 2, 86% of the included studies [35–39, 40] were judged to have low risk of bias for random sequence generation and the remaining study was judged to have unclear risk of bias [34]. For allocation concealment, 71% of the studies [36–39, 40] were judged as low risk of bias and the remaining studies were judged as unclear risk of bias [34–35]. Overall, 86% of studies [34–35, 37–39, 40] had a low risk of performance bias because either study participants could not be blinded given the nature of the intervention or only research personnel were blinded as it was not feasible to blind interventionists. The remaining study [36] was judged as unclear because there was insufficient evidence that the lack of blinding of participants influenced the outcomes.

Detection bias involves the blinding of outcome assessors [56]. All primary outcome measures were self-report measures, which are usually the most feasible types of measures to use in evaluating psychosocial interventions. Given that data collectors were blinded to the study group assignment in only 43% studies [36, 38, 40] judged as low risk of bias, about 57% of studies [34–35, 38–39] were judged as unclear. A total of 71% of studies [34–35, 37–39] were judged as low risk of attrition bias. The risk of attrition bias was unclear for the remainder of the studies [36, 30] because the number of drop outs was reported by study group but not with complete reasons. A total of 86% [34–35, 37–39, 40] of studies were judged as low risk of reporting bias and one study [36] was judged as unclear. For Other bias, all seven studies (100%) were judged as low risk of bias. Finally, the quality ratings for the seven included studies ranged from moderate to high. Six studies (86%) [34–37, 39, 40] were judged as moderate quality because these studies included unclear risks of bias. One study [38] was judged as very high quality since the risks of bias were low across all domains.

### Characteristics of study population

A total of 801 mothers and their partners (control group n = 385; intervention group n = 416) participated in all seven studies included in the meta-analysis. Two treatment studies and five prevention studies were included in the meta-analysis. The two treatment studies [34–35] only recruited mothers who met diagnostic criteria for Major Depressive Disorder during the postpartum period. The remaining five prevention studies included universal programs [36–39] and an indicated program for mothers at risk for developing depression [40]. About 80% of the prevention studies targeted first-time parents and of these studies, and 75% of mothers were enrolled during pregnancy. The remaining studies enrolled mothers within six months of the birth of the baby.

All studies reported maternal age and overall, the average age of mothers was 31.1 years old (SD = 4.92). The mean paternal age was only reported in two studies (M = 30.8 years old) but only one study reported the standard deviation. All couples were either married or in a committed relationship. Family annual income was reported in about 70% of studies and ranged from low to moderately high with the majority of participants having middle class annual incomes. About 85% of studies reported maternal education, it ranged from elementary school to college education with nearly all of mothers having at least a high school education. Parental race was only reported in three studies, and the majority of participants were White (91%). Since there was limited variability in demographic characteristics, subgroup analyses were not conducted for the primary outcomes.

### Quantitative results for primary outcomes

The overall findings for maternal depressive symptoms and family functioning, and the moderation analysis for maternal depression are presented in the following sections.

### Maternal depressive symptoms

**Fig 3:**
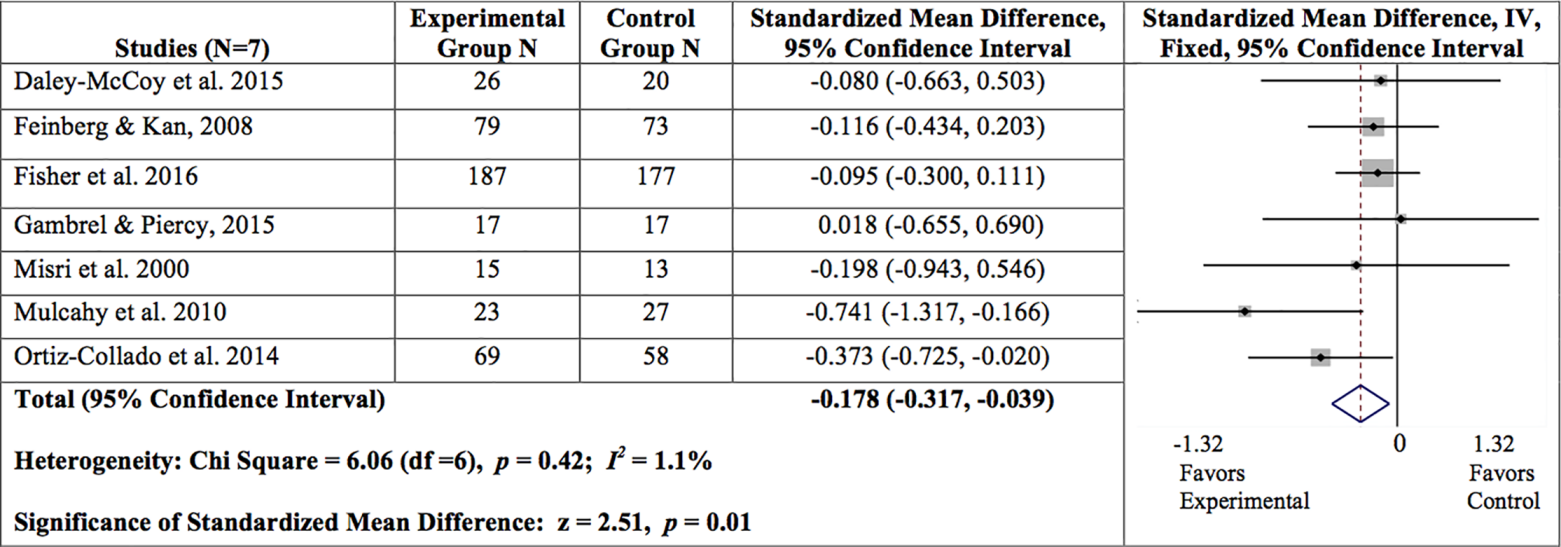
Summary of findings for maternal depressive symptoms at post-intervention.

For the maternal depressive symptoms, the overall findings for the meta-analysis that included all seven studies are presented in Fig 3. Overall, we found a statistically significant reduction in maternal depressive symptoms from baseline to the first follow up time point (SMD = -0.178, 95% CI = -0.317, -0.039; z = 2.51, *p* = .01; I^2^ = 1.1%).

A random effects model revealed significant associations for mothers who participated in an indicated preventive intervention (SMD = -0.373, 95% CI = -0.725, -0.020; z = 2.07, *p* = 0.04) and those who participated in treatment (SMD = -0.523, 95% CI = -1.045, -0.002; z = 1.97, *p* = 0.05) when compared to those who participated in universal prevention interventions (SMD = -0.093, 95% CI = -0.253, 0.068; z = 1.13, *p* = 0.26).

Two moderators were explored in the five prevention studies: dosage and level of family involvement. Level of family involvement was dichotomized using the mean percentage of sessions attended by family members (79% or fewer sessions = 2 studies; 80% of sessions or more = 3 studies). A fixed effects model revealed a significant association in that mothers of family members who attended at least 80% of the intervention sessions experienced a significantly greater decrease in depressive symptoms (SMD = -0.154, 95% CI = -0.309, 0.001; z = 1.94, *p* = 0.05, I^2^ = 0.0%) than those of family members who attended fewer intervention sessions (SMD = -0.038, 95% CI = -0.479, 0.402; z = 0.17, *p* = 0.87, I^2^ = 0.0%).

Dosage was measured by number of sessions and intervention duration in hours in the prevention studies (six or more sessions totaling at least 12 hours = 3 studies; five or fewer sessions under 12 hours = 2 studies). A fixed effects model revealed a trend in a reduction in depressive symptoms in mothers who attended sessions six or more sessions totaling at least 12 hours (SMD = -0.210, 95% CI = -0.429, 0.009; z = 1.88, *p* = 0.06; I^2^ = 0.0%) when compared to those with a lower dosage (SMD = -0.085, 95% CI = -0.282, 0.111; z = 0.85, *p* = 0.40; I^2^ = 0.0%).

### Family functioning

**Fig 4:**
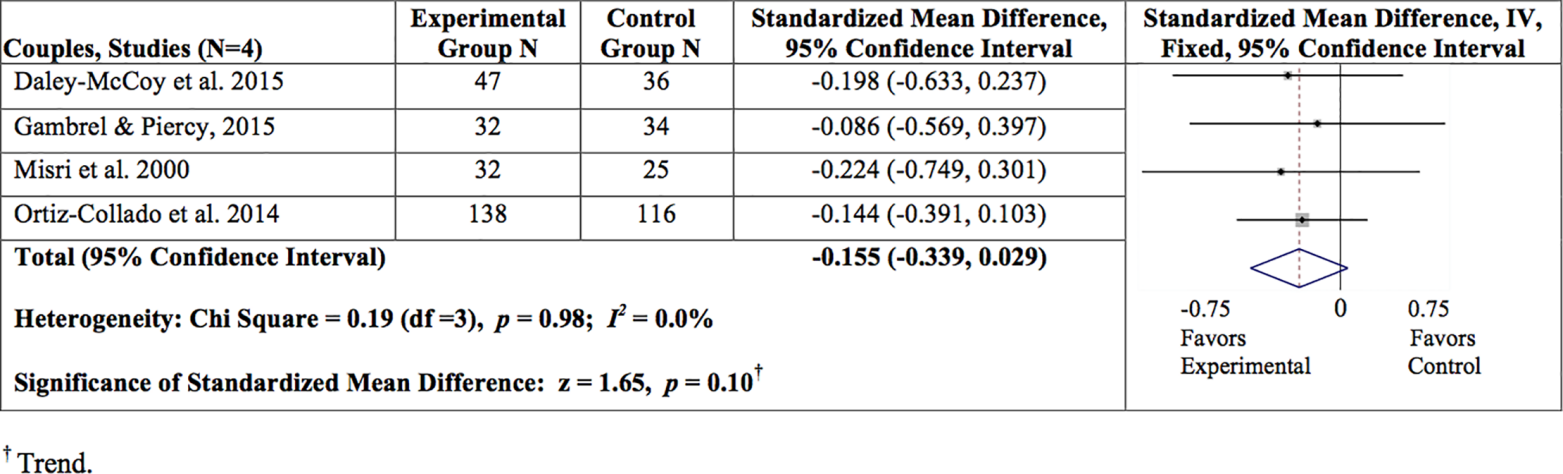
Summary of findings for family functioning at post-intervention. ^†^ Trend.

This outcome was evaluated at the couple-level, rather than the individual-level. A total of four studies (three prevention studies and one treatment study) were included in the analysis. The overall findings are presented for the meta-analyses for couples in Fig 4. Overall, the results revealed a trend in improving family functioning (SMD = -0.155, 95% CI = -0.339, 0.029; z = 1.65, *p* = .10; I^2^ = 0.0%). Since only four studies were included, moderation analysis was not possible for this outcome.

## Discussion

### Summary of evidence

The purpose of this systematic review and meta-analysis was to synthesize the available evidence on family therapeutic intervention impacts on reducing perinatal depressive symptoms and improving family functioning. Although all of the interventions targeted couples, the scope of the interventions primarily encompassed couple communication that pertained to parenting. For this reason, we did not define these systemic interventions as couple therapeutic interventions. Our findings showed that family therapeutic interventions significantly reduce perinatal depressive symptoms but the impact varies by intervention intensity (universal, indicated, or treatment). Overall, we found statistically significant reductions in perinatal depressive symptoms for mothers who participated in indicated preventive and treatment interventions. This finding is not surprising given that these subgroups of mothers showed greater reductions in depressive symptoms than did those who participated in universal interventions since their depressive symptoms were not that severe and there appeared to be little room for improvement. Our moderation analysis of the level of family involvement in the intervention was restricted to the five prevention studies. We were unable to do the moderation analysis for the treatment studies because there were only two studies, which is too few for a moderation analysis. As originally hypothesized, our analysis of level of family involvement showed that mothers of family members who attended at least 80% of the preventive intervention sessions experienced a statistically significant decrease in depressive symptoms when compared to those of family members who attended fewer sessions. This finding is consistent with the aim of these interventions to strengthen relationships, and positive relationships serve as a protective factor against depression [16]. Nonetheless, this finding should be interpreted with caution since we were only able to include five prevention studies in this analysis.

Dosage was measured by number of sessions attended by mothers and intervention duration in hours in the prevention studies. Our results for dosage only revealed a trend (*p* = .06) in reducing depressive symptoms for mothers who attended six or more sessions totaling at least 12 hours when compared to those who attended fewer sessions. However, it is possible that the inclusion of more studies could produce a significant result. For this reason, this finding should be interpreted with caution.

Furthermore, we found a trend (*p* = 0.10) in improving family functioning. We included four studies (three prevention studies and one treatment study) that measured this variable using responses from both partners. As previously mentioned, two prevention studies were excluded from this analysis because either the measures could not be standardized or the follow-up time points did not align with those of the other included studies. A treatment study was also excluded because the family functioning measure was only administered to mothers, and not their family members. It is possible that the smaller sample size contributed to the trend rather than a statistically significant result. For this reason, this result should be interpreted with caution.

### Limitations

There are some limitations in our study. First, the generalizability of our results may not be applicable to diverse populations. Given that the samples in the included studies lacked diversity (e.g., majority were middle class, primiparous mothers), there is a need for research with populations that vary by maternal age (e.g., adolescents vs adults), ethnicity and socioeconomic status. Furthermore, a key limitation of the literature reviewed on couple-based interventions for perinatal depression is the inclusion of only heterosexual couples. An important area for future research on this topic is the inclusion of same-sex couples. Second, we did not have access to the correlations for the baseline and follow-up outcome measures, which resulted in very conservative pooled standard deviations. Although statistical methods exist for imputing correlations, we were not comfortable using these methods and we assumed that baseline and follow-up measures were not correlated. If the baseline and follow-up outcome measures are correlated, then we most likely underestimated the significant effects of the intervention impacts on maternal depressive symptoms and family functioning. Finally, all of the measures for maternal depressive symptoms and family functioning were self-report and are subject to bias.

### Conclusions

Given the deleterious effects of perinatal depression on the family system [7, 9, 12], more research is needed on family therapeutic interventions that aim to prevent or treat perinatal depression. This area of research is innovative and the field is substantially growing. The limited number of included studies represents the few high quality controlled trials that have been conducted with this vulnerable population.

The research on family-based interventions for perinatal depression is still in the early stages of development. For this reason, we do not have data on the percentage of women who receive family therapy for the prevention or treatment of perinatal depression. Given that around 30% of adults with depression seek psychotherapy [62] and less than 30% of women who screen positive for perinatal depression attend a mental health visit (for psychotherapy or psychiatric medication) [63–65] with approximately 6% completing treatment in the United States [63,65], we suspect that a lower percentage of women with perinatal depression seek family therapy for two reasons.

First, stigma prevents many women with perinatal depression from seeking treatment because they are afraid of the consequences (e.g., Child Protective Services might think the mother is “crazy” and remove the baby from her care) [66]. Second, barriers may create difficulties in delivering and receiving these family therapeutic in real world settings. Barriers faced by providers may limit the use of family therapy interventions that aim to prevent or treat perinatal depression. For example, providers without family therapy training would need to incur costs for this type of training and possibly costs for supervision over an extended period of time, which may deter some providers from pursuing the needed education to deliver these types of interventions. Thus, the lack of available qualified providers may limit dissemination of these types of interventions. Furthermore, health insurance companies may vary in level of reimbursement for family therapy by provider type. In addition to barriers experienced by providers, families may encounter the following barriers: lack of childcare in postnatal populations, lack of family member availability due to time restrictions, and limited or no health insurance coverage for family therapy. In summary, the evidence that supports the use of family therapeutic interventions to prevent and treat perinatal depression described in our study should be considered in conjunction with potential barriers that interfere with implementation of these interventions and family receipt of these services.

The current study can serve as a catalyst for future research on the effectiveness of family therapeutic interventions that aim to prevent or treat perinatal depression and improve family functioning. Our primary recommendation is for future research to expand the existing knowledge with a wider variety of women (adolescents and adults with varied ethnicities and socioeconomic statuses) that may require different types and dosages of family therapeutic interventions. Furthermore, future research should include interventions that target the mother (pregnant and post-delivery) and her extended family members (e.g., grandparents, next of kin, adult siblings, etc.). Our study offers ample evidence to facilitate future research on family therapeutic interventions that aim to prevent or treat perinatal depression.

## Supporting information

S1 Table PRISMA checklist. (DOC)

## References

[1] American College of Obstetricians and Gynecologists. Screening for perinatal depression. Committee opinion no. 630. Obstet Gynecol. 2015;125:1268–1271. doi: 10.1097/01.AOG.0000465192.34779.dc 25932866 10.1097/01.AOG.0000465192.34779.dc

[2] Ashley JM, Harper BD, Arms-Chavez CJ, LoBello SG. Estimated prevalence of antenatal depression in the US population. Arch Womens Ment Health. 2016;19;395–400. doi: 10.1007/s00737-015-0593-1 26687691 10.1007/s00737-015-0593-1

[3] Ko JY, Rockhill KM, Tong VT, Morrow B, Farr SL. Trends in postpartum depressive symptoms– 27 States, 2004, 2008, 2012. MMWR Morbidity Mortal Weekly Report. 2017; 66:153–158. 10.15585/mmwr.mm6606a1 PMC5657855 28207685

[4] World Health Organization. Maternal mental health World Health Organization 2017 Retrieved from: http://www.who.int/mental_health/maternal-child/maternal_mental_health/en/

[5] Field T, Diego M, Hernandez-Reif M, Deeds O, Holder V, Schanberg S, et al Depressed pregnant black women have a greater incidence of prematurity and low birthweight outcomes. Infant Behav Dev. 2009;32:10–16. doi: 10.1016/j.infbeh.2008.09.005 19004502 10.1016/j.infbeh.2008.09.005 PMC2652730

[6] Dayan J, Creveuil C, Marks MN, Conroy S, Herlicoviez M, Dreyfus M, et al Prenatal depression, prenatal anxiety, and spontaneous preterm birth: A prospective cohort study among women with early and regular care. Psychosom Med. 2006;68:938–946. doi: 10.1097/01.psy.0000244025.20549.bd 17079701 10.1097/01.psy.0000244025.20549.bd

[7] Tronick E, Reck C. Infants of depressed mothers. Harvard Rev Psychiatry. 2009; 17:147–156. 10.1080/10673220902899714 19373622

[8] van Doesum KT, Hosman CM, Riksen-Walraven JM, Hoefnagels C. Correlates of depressed mothers’ sensitivity toward their infants: The role of maternal, child, and contextual characteristics. J Am Acad Child Adolesc Psychiatry. 2007; 46: 747–756. doi: 10.1097/CHI.0b013e318040b272 17513987 10.1097/CHI.0b013e318040b272

[9] Easterbrooks MA, Bartlett JD, Raskin M, Goldberg J, Contreras MM, Kotake C, et al Limiting home visiting effects: maternal depression as a moderator of child maltreatment. Pediatrics. 2013;132(Supp 2):S126–33. 24187114 10.1542/peds.2013-1021K

[10] Dennis CL. Psychosocial interventions for the treatment of perinatal depression. Best Pract Res Clin Obstet Gynaecol. 2014;28:97–111. doi: 10.1016/j.bpobgyn.2013.08.008 24070822 10.1016/j.bpobgyn.2013.08.008

[11] Dennis CL, Dowswell T. Psychosocial and psychological interventions for preventing postpartum depression. Cochrane Database of Systematic Reviews 2013;2: CD001134. 10.1002/14651858.CD001134.pub3 PMC11936315 23450532

[12] Barlow J, Bennett C, Midgley N, Larkin SK, Wei Y. Parent-infant psychotherapy for improving parental and infant mental health. Cochrane Database of Systematic Reviews 2015;1:CD010534 doi: 10.1002/14651858.CD010534.pub2 25569177 10.1002/14651858.CD010534.pub2 PMC8685508

[13] Letourneau NL, Dennis CL, Cosic N, Linder J. The effect of perinatal depression treatment for mothers on parenting and child development: A systematic review. Depress Anxiety. 2017; 34:928–966. doi: 10.1002/da.22687 28962068 10.1002/da.22687

[14] Lancaster CA, Gold KJ, Flynn HA, Yoo H, Marcus SM, Davis MM. Risk factors for depressive symptoms during pregnancy: a systematic review. Am J Obstet Gynecol. 2010;202:5–14. doi: 10.1016/j.ajog.2009.09.007 20096252 10.1016/j.ajog.2009.09.007 PMC2919747

[15] Field T, Diego M, Hernandez-Reif M. Prenatal depression effects and interventions: A review. Infant Behav Dev. 2010;33:409–418. doi: 10.1016/j.infbeh.2010.04.005 20471091 10.1016/j.infbeh.2010.04.005 PMC2933409

[16] Dennis CL, Ross L. Women's perceptions of partner support and conflict in the development of postpartum depressive symptoms. J Adv Nurs. 2006;56:588–99. doi: 10.1111/j.1365-2648.2006.04059.x 17118038 10.1111/j.1365-2648.2006.04059.x

[17] Johnstone SJ, Boyce PM, Hickey AR, Morris-Yatees AD, Harris MG. Obstetric risk factors for postnatal depression in urban and rural community samples. Aust N Z J Psychiatry. 2001;35:69–74. doi: 10.1046/j.1440-1614.2001.00862.x 11270460 10.1046/j.1440-1614.2001.00862.x

[18] Morse CA, Buist A, Durkin S. First-time parenthood: influences on pre- and postnatal adjustment in fathers and mothers. J Psychosom Obstet Gynaecol. 2000;21:109–20. 10994183 10.3109/01674820009075616

[19] Leathers SJ, Kelley MA. Unintended pregnancy and depressive symptoms among first time mothers and fathers. Am J Orthopsychiatry. 2000;70:523–31. 11086530 10.1037/h0087671

[20] Feinberg ME. The Internal Structure and Ecological Context of Coparenting: A Framework for Research and Intervention. Parent Sci Pract. 2003;3(2):95–131. doi: 10.1207/S15327922PAR0302_01 21980259 10.1207/S15327922PAR0302_01 PMC3185375

[21] Zelkowitz P, Schinazi J, Katofsky L, Saucier JF, Valenzuela M, Westreich R, et al Factors associated with depression in pregnant immigrant women. Transcultural Psychiatry. 2004;41:445–64. doi: 10.1177/1363461504047929 15709645 10.1177/1363461504047929

[22] O’Hara MW. Social support, life events and depression during pregnancy and the puerperium. Arch Gen Psychiatry. 1986;43:569–73. 3707289 10.1001/archpsyc.1986.01800060063008

[23] Morinaga K, Yamauchi T. Childbirth and changes of women’s social support network and mental health. Shinrigaku Kenkyu. 2003;74:412–419. 15029757 10.4992/jjpsy.74.412

[24] Logsdon MC, Usui W. Psychosocial predictors of postpartum depression in diverse groups of women. West J Nurs Res. 2001;23:563–574. doi: 10.1177/019394590102300603 11569330 10.1177/019394590102300603

[25] Viinamaki H, Niskanen L, Pesonen P, Saarikoski S. Evolution of postpartum mental health. J Psychosom Obstet Gynaecol. 1997;18: 213–219. 9304542 10.3109/01674829709080690

[26] Zhang R, Chen Q, Li Y. Study for the factors related to postpartum depression. Chinese J Obstet Gynecol. 1999;34: 231–233. 11326923

[27] Patel V, Rodrigues M, DeSouza N. Gender, poverty, and postnatal depression: a study of mothers in Goa, India. Am J Psychiatry. 2002;159:43–47. doi: 10.1176/appi.ajp.159.1.43 11772688 10.1176/appi.ajp.159.1.43

[28] Fisher JR, Feekery CJ, Rowe-Murray HJ. Nature, severity and correlates of psychological distress in women admitted to a private mother-baby unit. Paediatr Child Health. 2002;38:140–145. 10.1046/j.1440-1754.2002.00723.x 12030994

[29] Bateson G, Jackson DD, Haley J, Weakland J. Toward a theory of schizophrenia. Behavioral Sci. 1956;1:251–264.

[30] von Bertalanffy L. General system theory: Foundations, development, applications New York: George Braziller 1968.

[31] Diamond GS, Wintersteen MB, Brown GK, Diamond GM, Gallop R, Shelef K, et al Attachment-based family therapy for adolescents with suicidal ideation: A randomized controlled trial. Am Acad Child Adolesc Psychiatry. 2010;49(2):122–131. 10.1097/00004583-201002000-00006 20215934

[32] Jensen TK, Holt T, Ormhauga SM, Egeland K, Granly L, Hoaas LC, Hukkelberg SS, et al A randomized effectiveness study comparing trauma-focused cognitive behavioral therapy with therapy as usual for youth. J Clin Child Adolesc Psychol. 2013;0(0):1–14. 10.1080/15374416.2013.822307 PMC4037845 23931093

[33] Azrin NH, Acierno R, Kogan ES, Donohue B, Besalel VA, McMahon PT. Follow-up results of supportive versus behavioral therapy for illicit drug use. Behav Res Thera. 1996;34:41–46. 10.1016/0005-7967(95)00049-4 8561763

[34] Misri S, Kostaras X, Fox D, Kostaras D. The impact of partner support in the treatment of postpartum depression. Can J Psychiatry. 2000;45:554–558. doi: 10.1177/070674370004500607 10986574 10.1177/070674370004500607

[35] Mulcahy R, Reay RE, Wilkinson R, Owen C. A randomised control trial for the effectiveness of group interpersonal psychotherapy for postnatal depression. Arch Womens Ment Health. 2010;13:125–139. doi: 10.1007/s00737-009-0101-6 19697094 10.1007/s00737-009-0101-6

[36] Daley-McCoy C, Rogers M, Slade P. Enhancing relationship functioning during the transition to parenthood: A cluster-randomised controlled trial. Arch Womens Ment Health. 2015;18:681–692. doi: 10.1007/s00737-015-0510-7 25663309 10.1007/s00737-015-0510-7

[37] Fisher J, Rowe H, Wynter K, Tran T, Lorgelly P, Amir LH, et al Gender informed, psychoeducational programme for couples to prevent postnatal common mental disorders among primiparous women: Cluster randomised controlled trial. BMJ. 2016;6:e009396 doi: 10.1136/bmjopen-2015-009396 26951210 10.1136/bmjopen-2015-009396 PMC4785308

[38] Feinberg ME, Kan ML. Establishing Family Foundations: Intervention effects on coparenting, parent/infant well-being, and parent-child relations. J Fam Psychol. 2008;22:253–263. doi: 10.1037/0893-3200.22.2.253 18410212 10.1037/0893-3200.22.2.253 PMC3178882

[39] Gambrel LE, Piercy F. Mindfulness-based relationship education for couples expecting their first child–Part 1: A randomized mixed-methods program evaluation. J Marital Fam Thera. 2015;1:5–24. 10.1111/jmft.12066 24433518

[40] Ortiz Collado MA, Saez M, Favrod J, Hatem M. Antenatal psychosomatic programming to reduce postpartum depression risk and improve childbirth outcomes: A randomized controlled trial in Spain and France. BMC Pregnancy Childbirth. 2014;14:22 doi: 10.1186/1471-2393-14-22 24422605 10.1186/1471-2393-14-22 PMC3898772

[41] Pinquart M, Teubert D. A meta-analytic study of couple interventions during the transition to parenthood. Fam Relat. 2010;59:221–231.

[42] Suto M, Takehara K, Yamane Y, Ota E. Effects of prenatal childbirth education for partners of pregnant women on paternal postnatal mental health and couple relationship: A systematic review. J Affect Disord. 2017;210:115–121. doi: 10.1016/j.jad.2016.12.025 28024222 10.1016/j.jad.2016.12.025

[43] Morrell CJ, Sutcliffe P, Booth A, Stevens J, Scope A, Stevenson M, et al A systematic review, evidence synthesis and meta-analysis of quantitative and qualitative studies evaluating the clinical effectiveness, the cost-effectiveness, safety and acceptability of interventions to prevent postnatal depression. Health Technol Assess. 2016;20(37). doi: 10.3310/hta20370 27184772 10.3310/hta20370 PMC4885009

[44] Lever Taylor B, Cavanagh K, Strauss C. The effectiveness of mindfulness-based interventions in the perinatal period: A systematic review and meta-analysis. PLoS ONE. 2016;11(5):e0155720 doi: 10.1371/journal.pone.0155720 27182732 10.1371/journal.pone.0155720 PMC4868288

[45] Pilkington PD, Whelan TA, Milne LC. A review of partner-inclusive interventions for preventing postnatal depression and anxiety. Clin Psychologist. 2015;63–75.

[46] Claridge AM. Efficacy of systemically oriented psychotherapies in the treatment of perinatal depression: A meta-analysis. Arch Womens Ment Health. 2014;17:3–15. doi: 10.1007/s00737-013-0391-6 24240636 10.1007/s00737-013-0391-6

[47] Werner E, Miller L, Osborne LM, Kuzava S, Monk C. Preventing postpartum depression: Review and recommendations. Arch Womens Ment Health. 2014;18:41–60. doi: 10.1007/s00737-014-0475-y 25422150 10.1007/s00737-014-0475-y PMC4308451

[48] Stevenson MD, Scope A, Sutcliffe PA, Booth A, Slade P, Parry G, et al Group cognitive behavioural therapy for postnatal depression: a systematic review of clinical effectiveness, cost-effectiveness and value of information analyses. Health Technol Assess. 2010;14(44). doi: 10.3310/hta14440 20863477 10.3310/hta14440

[49] Cluxton-Keller F, Riley AW, Noazin S, Umoren MV. Clinical effectiveness of family therapeutic interventions embedded in general pediatric primary care settings for parental mental health: A systematic review and meta-analysis. Clin Child Fam Psychol Rev. 2015;18:395–412. doi: 10.1007/s10567-015-0190-x 26377209 10.1007/s10567-015-0190-x

[50] Higgins JPT, Green S, eds. Cochrane handbook for systematic reviews of interventions Version 5.1.0 [updated March 2011]. The Cochrane Collaboration 2011 Available from www.cochrane-handbook.org

[51] Moher D, Liberati A, Tetzlaff J, Altman DG, The PRISMA Group. Preferred Reporting Items for Systematic Reviews and Meta-Analyses: The PRISMA Statement. PLoS Med 2009;6(6):e1000097. 19621072 10.1371/journal.pmed.1000097 PMC2707599

[52] Guyatt GH, Oxman AD, Kunz R, Atkins D, Brozek J, Vist G, et al GRADE guidelines: 2. Framing the question and deciding on important outcomes. J Clin Epidemiol. 2011;64:395–400. doi: 10.1016/j.jclinepi.2010.09.012 21194891 10.1016/j.jclinepi.2010.09.012

[53] Higgins JPT, Altman DG, Gøtzsche PC, Jüni P, Moher D, Oxman AD, et al The Cochrane Collaboration’s tool for assessing risk of bias in randomised trials. BMJ. 2011;343:d5928 doi: 10.1136/bmj.d5928 22008217 10.1136/bmj.d5928 PMC3196245

[54] Higgins JPT, Deeks JJ, Altman DG. Special topics in statistics In: Higgins JPT, Green S, eds. Cochrane handbook for systematic reviews of interventions Version 5.1.0 (updated March 2011). The Cochrane Collaboration 2011 Available from www.cochrane-handbook.org

[55] Schünemann H, Brożek J, Guyatt G, Oxman A, eds. GRADE handbook for grading quality of evidence and strength of recommendations The GRADE Working Group, 2013 Available from www.guidelinedevelopment.org/handbook

[56] Higgins JPT, Altman DG, Sterne JAC. Assessing risk of bias in included studies In: Higgins JPT, Green S, eds. Cochrane handbook for systematic reviews of interventions Version 5.1.0 (updated March 2011). The Cochrane Collaboration 2011 Available from www.cochrane-handbook.org

[57] Review Manager (RevMan) [Computer program]. Version 5.3. Copenhagen: The Nordic Cochrane Centre, The Cochrane Collaboration, 2014.

[58] Stata Corporation. Stata/SE 15.1. College Station, TX: Stata Corporation.

[59] Deeks JJ, Higgins JPT, Altman DG. Analysing data and undertaking meta-analyses In: Higgins JPT, Green S, eds. Cochrane handbook for systematic reviews of interventions Version 5.1.0 (updated March 2011). The Cochrane Collaboration Available from www.cochrane-handbook.org

[60] Moher D, Liberati A, Tetzlaff J, Altman DG, The PRISMA Group. Preferred Reporting Items for Systematic Reviews and Meta-Analyses: The PRISMA Statement. PLoS Med. 2009;6(7): e1000097 doi: 10.1371/journal.pmed.1000097 19621072 10.1371/journal.pmed.1000097 PMC2707599

[61] Epstein NB, Zheng L. Cognitive-behavioral couple therapy. Curr Opin Psychol. 2017;13:142–147. doi: 10.1016/j.copsyc.2016.09.004 28813285 10.1016/j.copsyc.2016.09.004

[62] Olfson M, Blanco C, Marcus SC. Treatment of adult depression in the United States. JAMA Intern Med. 2016;176(10):1482–1491. doi: 10.1001/jamainternmed.2016.5057 27571438 10.1001/jamainternmed.2016.5057

[63] Smith MV, Howell H, Wang H, Poschman K, Yonkers KA.Success of mental health referral among pregnant and postpartum women with psychiatric distress. Gen Hosp Psychiatry. 2009;31(2):155–62. doi: 10.1016/j.genhosppsych.2008.10.002 19269536 10.1016/j.genhosppsych.2008.10.002 PMC2867091

[64] Marcus SM, Flynn HA, Blow FC, Barry KL. Depressive symptoms among pregnant women screened in obstetrics settings. J Womens Health (Larchmt). 2003;12(4):373–80. 12804344 10.1089/154099903765448880

[65] Rowan P, Greisinger A, Brehm B, Smith F, McReynolds E. Outcomes from implementing systematic antepartum depression screening in obstetrics. Arch Womens Ment Health. 2012;15(2):115–20. doi: 10.1007/s00737-012-0262-6 22382279 10.1007/s00737-012-0262-6

[66] Zauderer C. Postpartum depression: How childbirth educators can help break the silence. J Perinat Educ. 2009;18(2):23–31. doi: 10.1624/105812409X426305 20190853 10.1624/105812409X426305 PMC2684038

